# Prevalence and influencing factors of self-medication during the COVID-19 pandemic in the Arab region: a multinational cross-sectional study

**DOI:** 10.1186/s12889-023-15025-y

**Published:** 2023-01-27

**Authors:** Aya Elsayed Abdelwahed, Mostafa Mahmod Abd-elkader, Amany Mahfouz, Mohammed Osama Abdelmawla, Marwa Kabeel, Amr Gabr Elkot, Mohamed Rabiea Hamad, Rahma Abd Elfattah Ibrahim, Marwa M. I. Ghallab, Collaborators group, Jaafar D. Al-Dabagh, Ali R. Abdulabbas, Mohammed A. A. Osman, Mazen M. O. Barakat, Maya M. Abdelwahab, Israa Al-fayyadh, Tharaa Khairy, Mabrouka Salmi, Asmaa R. A. Elsokary, Tayba Mugibel, Batool A. A. Abuelsamen, Mostafa M. Naguib, Yassen M. Alothman, Omar Q. Heih, Ramadan A. Farahat, Imene Maallem, Nagy N. Mohamed, Areej M. Al-Mamari, Fatima H. Bin Yahya, Maryam O. Al Amodi, Ghada A. El-Shafey, Esra E. Elkhoja, Salma A. Shawky, Salma Saleh, Fawzy Shendy, Tharaa Tobba, Omar T. Elnewishy, Tima Al Shammaa, Wisam M. Alismaeil, Aiman S. Gmzawi, Rama N. Basheer, Bashar H. Hassan, Eman S. Barhoom, Areej Abumazen, Majd AL-lala, Maathir I. Alfarsi, Lujain B. S. Laik, Muath M. Mohammed, Noof K. B. Abo Baker, Marwan E. Mohamed, Hassan F. H. Elsayed, Waad N. Almanaseer, Yasmine R. Albalushi, Hawwa Altaeb, Reem J. Husseiny, Mohammed Orief, Fatima Alaidaros, Hajar Fawzy, Moh. Mah. Fadelallah Eljack, Nouran Hamza

**Affiliations:** 1 Faculty of Medicine, Kafrelsheikh University, Kafr El-Sheikh, Egypt; 2 Department of Medical Parasitology, Faculty of Medicine, KafrElsheikh University, Kafr El-Shaikh, Egypt; 3 Medical Agency for Research and Statistics, Giza, Egypt

**Keywords:** Self-medication, Arab, COVID-19, Drugs, Coronavirus, Antibiotic

## Abstract

**Background:**

The novel coronavirus pandemic (COVID-19) has begun with a wave of misinformation and fear of infection. This may have led people to self-medicate inappropriately. The World Health Organization describes self-medication (SM) as utilizing medicines to relieve symptoms or health conditions without consulting a physician. Inappropriate drug use is a burden on both health resources and patient health in the Arab region. This study aimed to detect the prevalence and influencing factors of self-medication among the general Arab population during the COVID-19 pandemic.

**Methods:**

A multinational cross-sectional study was conducted among the general population of ten Arab countries from early August to late October 2021. Participants aged 18 years or older could join the study via social media platforms. A convenience sampling technique was used. A developed and validated web-based questionnaire was used to collect data on self-medication practice, associated influencing factors, information sources, commonly used medications, and commonly treated conditions. Descriptive, univariate, and multivariate regression analyses were applied using IBM SPSS v 26 and R v 4.0.0 software.

**Results:**

A total of 8163 participants completed the questionnaire, and 518 participants were excluded from the analysis due to inconsistencies in their data. Almost two-thirds (62.7%) of participants reported practicing self-medication during the COVID-19 pandemic. At the country level, Egypt had the highest prevalence of self-medication practice (72.1%), while Palestine had the lowest prevalence (40.4%). The most commonly used drugs were analgesics, antipyretics, and vitamins (86, 65.1, and 57.1%, respectively), while antitussives and antibiotics scored 47.6 and 43.3%, respectively. Experience with similar health conditions (74.6%) and urgency of the problem (47.2%) were the most frequent factors that led to self-medication. Additionally, 38.2% of the self-medicated participants (SMPs) used drugs as prophylaxis against COVID-19. Pharmacist consultation was the most common source of information about self-medication (66.7%). Multivariate analysis showed that predictors of self-medication were older age (*p* = 0.008), presence of chronic illness (*p* = 0.015), and having monthly income or medical insurance that does not cover the treatment cost (*p* = 0.001, *p* < 0.001, respectively).

**Conclusion:**

Self-medication is considered a common practice across the Arab population. It is necessary to regulate policies and raise awareness among the public about self-medication.

**Supplementary Information:**

The online version contains supplementary material available at 10.1186/s12889-023-15025-y.

## Background

From Wuhan city in Central China’s Hubei Province, the novel coronavirus disease (COVID-19) emerged in December 2019 [1]. Subsequently, it rapidly spread to other parts of the world with rising morbidity and mortality rates [2, 3]. Then, the World Health Organization (WHO) declared it a global pandemic in March 2020 [4]. In response to this emergency, governments globally had to adopt restrictive measures such as lockdowns and social distancing. This has made access to healthcare services very challenging and has led many people to self-medicate [5]. Other factors that could have increased the practice of self-medication (SM) were people’s fear of catching the virus, the absence of definitive treatment for COVID-19, and the absence of COVID-19 vaccines during the early period of the pandemic crisis [6–8]. Furthermore, the misinformation circulating on social platforms has created a massive hysteria about the prevention and treatment of COVID-19; this infodemic is likely to have further increased the practice of self-medication (SM) among the general population [8, 9].

The WHO defines self-medication as “the use of medicinal products by the consumer to treat self-recognized disorders or symptoms, or the intermittent or continued use of a medication prescribed by a physician for chronic or recurring diseases or symptoms. In practice, it also includes the use of the medication of family members, especially where the treatment of children or the elderly is involved” [10]. Self-medication (SM) is a frequent behavior worldwide. Several studies have determined that the prevalence of self-medication before the COVID-19 pandemic ranged from 11.2 to 93.7% based on the studied population and country [11–20]. Google searches about SM have increased since the declaration of the COVID-19 pandemic [21]. This would imply a rise in interest in persons looking for information about self-medication to treat various illnesses.

The appropriate use of self-medication has a positive influence on patients and healthcare systems [22]. It allows patients to manage their health status, thus raising self-empowerment. It is convenient to prevent and relieve minor conditions. At the healthcare system level, it decreases wasting medical resources on minor conditions and reduces the load on healthcare services [22, 23]. This has been useful at times when healthcare systems were overwhelmed by the COVID-19 situation [22]. Furthermore, it can have economic benefits for patients, healthcare systems, and third-party payers such as governments or insurance companies [24]. However, several studies have found that improper self-medication practices can put patients at risk for serious complications such as adverse reactions, comorbidities, and increasing antimicrobial resistance, which is currently a global challenge [25–29]. The most common self-administered medications include painkillers, antipyretics, antidiarrheals, cough syrups, vitamins, sleeping pills, antibiotics, herbals, and home remedies [30]. Information about SM may come from family, friends, pharmacists, previous prescriptions, and the media [31]. Previous studies have shown that self-medication patterns differ from one population to another and are affected by several sociodemographic factors, including age, gender, income, education level, and medical knowledge [32–36].

The main objective of this study was to assess the prevalence and influencing factors of self-medication in general among the Arab general population during the COVID-19 pandemic. It also aimed to determine the most commonly self-prescribed drugs and sources of information about SM. The results of this study will inform healthcare policymakers about the proper measures that would regulate SM practices in the Arab region.

## Methods

### Study design and population

A multinational cross-sectional study was conducted among the general population of 10 Arab countries (Algeria, Egypt, Iraq, Jordan, Palestine, Saudi Arabia, Sudan, Oman, Syria, and Yemen) using a web-based questionnaire. Arab adults aged 18 years old or above were eligible to participate. Respondents who did not complete the questionnaire participated in the pilot phase or who responded to the questionnaire within less than 1 min were excluded.

### Sample size calculation

Epi Info software version 7.2.4.0 was used to estimate the sample size. It was 384 for each country at a confidence level of 95%. The investigators assumed that 50% of the population would practice self-medication with a 5% margin error.

### Questionnaire development and measurement

A questionnaire was designed based on previous similar studies and literature reviews (as shown in Additional file 1) [37–40]. It was prepared in English and then translated into Arabic. We applied back translation into English again and made sure about the equivalence of the meaning. After that, a pilot study was conducted to help reveal any ambiguity before mass spreading. Three experts were reached to evaluate the clarity, comprehensiveness, and relevance of the questions to the study objectives. During the first 10 days of July 2021, participants were recruited for the pilot study to rate the clarity and relevance of the questions to the study objectives, and a total of 592 participants completed the Arabic version of the questionnaire, while 77 participants completed the questionnaire in English. The questionnaire was then edited based on the opinions of experts and the feedback from participants. The pilot study participants were not included in the final analysis.

Cronbach’s alpha test was also used to measure the reliability of the questions for both versions. The Arabic version achieved 0.708 after removing two questions, while the English version scored 0.502. The English version of the questionnaire was not used for data collection due to its poor reliability and low response rate.

The final version of the questionnaire consisted of three sections. The first section discussed information related to demographics such as age, gender, occupation, educational level, and living area. The second section assessed the prevalence of SM, which was measured by asking “Have you ever taken any medicine/drug without consulting a qualified physician?” with responses of “never which was considered NO, and coded as 0”,and “once, seldom (2-3 times a year), sometimes (once every few months), often (once every few weeks), always which were all considered Yes for SM and coded as 1). The third section included questions related to the most common drug used, for what conditions, influencing factors, and sources of information about self-medication; these were multiple choice questions, and each choice had 2 possible answers: “yes“ and “no“. each was coded as “1” and “0”, respectively. Additionally, the consumption rate of the self-medication practice was assessed in the third section (decreased, remained unchanged, or increased during the pandemic). These options were coded from 1 to 3 in order. All questions were mandatory to be answered if the participants reported that they practiced self-medication. On the other hand, the questionnaire ended automatically in the first section if a participant replied that they had never practiced self-medication.

### Data collection

Social media platforms were used to recruit collaborators from 12 Arab countries who helped collect the necessary data on self-medication from the target population. A convenience sampling technique was used to recruit respondents from August 1 to October 30, 2021, and questionnaires were distributed via social media platforms to collect data on self-medication. For countries that had given their ethical approval to conduct this study, data were also collected offline to achieve the target sample size. Ten countries accomplished the required sample size (Algeria, Egypt, Iraq, Jordan, Palestine, Saudi Arabia, Sudan, Oman, Syria, and Yemen). Offline collected data were transferred to a Microsoft Excel sheet for organization prior to analysis.

### Analysis

The data were organized in a Microsoft Excel sheet and then exported to IBM SPSS version 26 to conduct descriptive analysis for demographics and other study variables.

The “age” variable was the only numerical one and was tested for normality using a boxplot and Shapiro test that both revealed its skewness so was described in median (minimum-maximum). A univariate and multivariate logistic regression model was used to determine the likelihood of the included factors in terms of crude and adjusted odds ratio. The dependent variable was self-medication practice during the pandemic, which was considered binomial (yes/no), and the independent variables included were selected from the very beginning based on the literature. A hierarchy approach was applied, and Hosmer–Lemeshow resulted in a *p* value of 0.658, indicating the goodness of model fit.

## Results

**Fig. 1:**
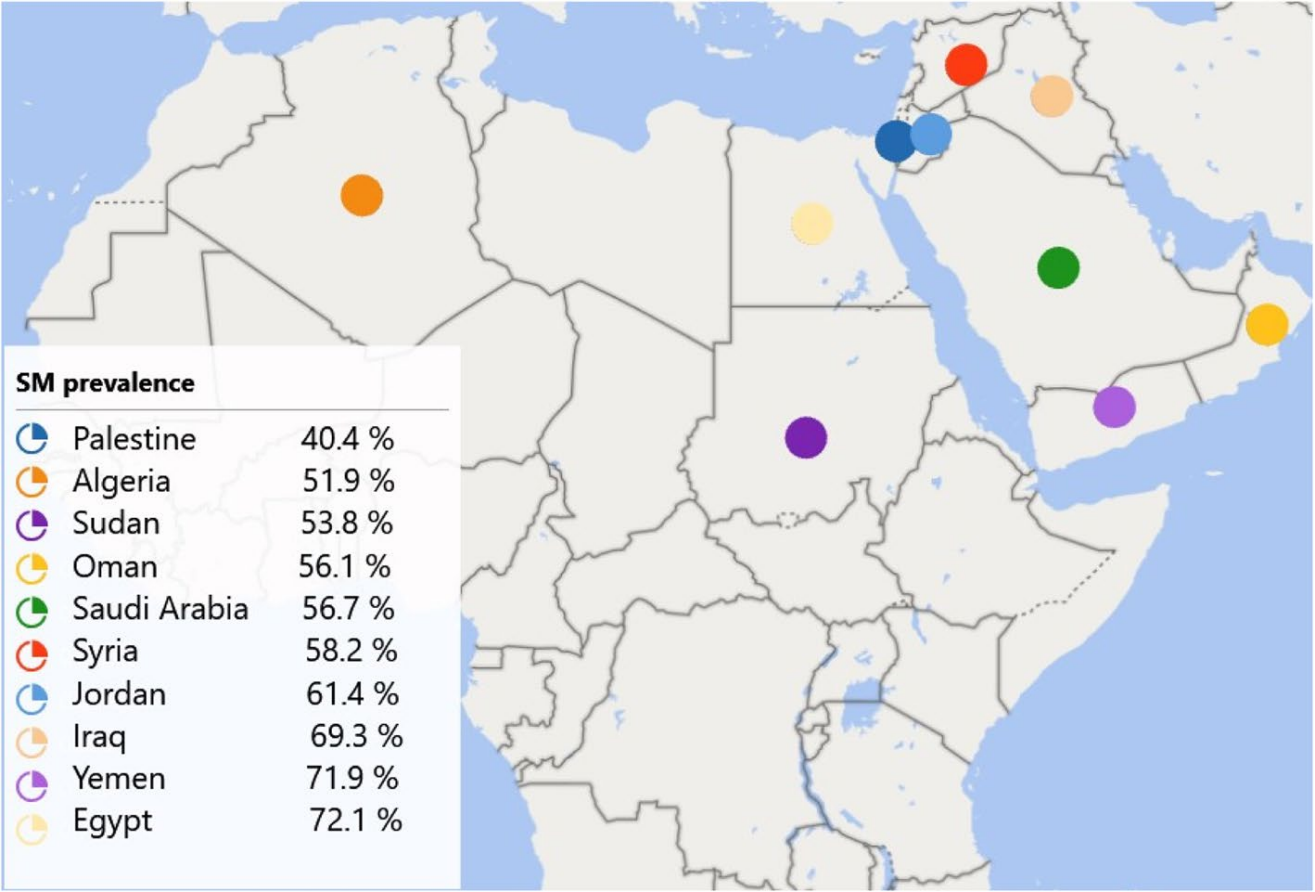
Self-medication practice across the different countries

In total, 8163 participants completed the questionnaire. Egypt had the highest response rate (28.4%), while Oman had the lowest response rate (5%). Data from 518 participants were excluded from the analysis due to their inconsistency. The final analysis included data from 7645 respondents. Table 1 shows the demographic information of the participants. Most of the respondents were young adults, with a median age of 22 years, ranging from 18 to 93 years. Of them, 2638 participants (34.5%) were males, while 5007 participants (65.5%) were females. The great majority of respondents were well educated with a university education (80.5%), lived in an urban area (76.8%), were in the medical field (42.1%), did not have medical insurance (58.1%), and had a monthly income that could cover the treatment cost (63.1%) (Table 1). Almost two-thirds of the participants (62.7%) reported using drugs without a doctor’s supervision. The prevalence differs from one country to another. Egypt had the greatest prevalence (72.10%), while Palestine had the lowest prevalence of SM (40.40%) (Fig. 1).

**Table 1:** Demographic and characteristics of respondents (*n* = 7645)

Variable	Category	n (%)
Gender	Male	2638 (34.5%)
	Female	5007 (65.5%)
Area of residence	Rural	1774 (23.2%)
	Urban	5871 (76.8%)
Education	Uneducated	61 (0.8%)
	Preuniversity education	711 (9.3%)
	University education (under and postgraduates)	6154 (80.5%)
	Higher education	719 (9.4%)
Occupation^a^	Governmental employee	765 (10.0%)
	Nongovernmental employee	711 (9.3%)
	Freelancer	612 (8.0%)
	Not working	1055 (13.8%)
	Medical field related (doctor or student)	3219 (42.1%)
	Nonmedical student	1575 (20.6%)
	Others	54 (0.7%)
Medical insurance	No, I don’t have medical insurance	4440 (58.1%)
	Yes, but it does not cover the treatment cost	1773 (23.2%)
	Yes, and it covers the treatment cost	1421 (18.6%)
Monthly income	prefer not to say	879 (11.5%)
	no, it does not cover the cost	443 (5.8%)
	Yes, but it does not cover the treatment cost	1498 (19.6%)
	Yes, and it covers the treatment cost	4823 (63.1%)
Age^b^ (years)	22 (18–93)	

^a^Multiresponse question. Medical insurance has 7642 responses, monthly income has 7643 responses, and the rest is missing

^b^Age is calculated as the median (minimum-maximum)

Pain killers were the most consumed drugs (86.0%), followed by fever-relieving pills, vitamins, anticoagulants, and antibiotics (65.1, 57.1, 47.6, and 43.3%, respectively) (Table 2).

**Table 2:** Medications used for self-medication (*N* = 4793)^b^

Medication type^a^	n (%)
Pain killers	4122 (86.0%)
Fever relieving meds	3120 (65.1%)
Vitamins	2737 (57.1%)
Antitussives	2281 (47.6%)
Herbal	2147 (44.8%)
Antibiotics	2075 (43.3%)
Anti-allergic meds	1332 (27.8%)
Pills for indigestion	1016 (21.2%)
Others	724 (15.1%)
Sedatives	484 (10.1%)
Sleeping pills	422 (8.8%)
Birth control pills	177 (3.7%)

^a^Multiple responses

^b^166 responses were omitted due to inconsistency of the data

People tended to self-medicate when they had headaches (85.4%), flu common-cold and cough together (67.4%), and pain elsewhere (56.0%) (Table 3).

**Table 3:** Health conditions for which self-medication is practiced (*N* = 4793)

Condition category^a^	n (%)
Headache	4093 (85.4%)
Flue-Common Cold-Cough	3230 (67.4%)
Pain elsewhere	2684 (56.0%)
Fever	2296 (47.9%)
Diarrhea-Vomiting	1821 (38.0%)
Allergy	1198 (25.0%)
Others	700 (14.6%)
Insomnia	685 (14.3%)

^a^Multiple responses

Experience with similar conditions and urgency of the case were the most common factors leading to SM (74.6 and 47.2%, respectively). A considerable number of participants practiced SM due to fear of infection (41.6%), while 38.2% were taking drugs as a prophylaxis against COVID-19 (Table 4).

**Table 4:** Reasons behind self-medication practice (*N* = 4793)

Reason^a^	n (%)
Experience of similar condition	3576 (74.6%)
Urgency	2262 (47.2%)
Fear of infection	1994 (41.6%)
Prophylaxis against covid-19	1831 (38.2%)
Prior commitments	1582 (33%)
Cost of the consultation	1146 (23.9%)
Others	863 (18%)
Unavailability of transport	479 (10%)

^a^Multiple responses

Pharmacist consultation was the most common source of information about self-prescribed medications (66.7%), followed by academic experience and prior prescription of the participant with the same frequency (50.1%) (Table 5). The consumption rate of 19.7% of people who practice self-medication increased during the pandemic, while 63.9% remained unchanged.

**Table 5:** Source of information about medications (*N* = 4793)

^a^Source of information	n (%)
Pharmacist consultation	3197 (66.7%)
My own academic knowledge	2401 (50.1%)
Prior prescriptions of my own	2401 (50.1%)
Friends and relatives	1745 (36.4%)
Other medical and pharmaceutical students	1548 (32.3%)
Internet	1447 (30.2%)
Previous prescriptions of others	767 (16.0%)
Others	633 (13.2%)
Advertisements	259 (5.4%)

^a^Multiple responses

In a univariate model, significantly increased odds of self-medication (OR: 1.02; 95% CI: 1.01–1.02, *p* < 0.001) were observed with increasing age. Additionally, there was a significantly increased odds of self-medication if the participant was a governmental employee (OR: 1.47; 95% CI: 1.25–1.73, *p* < 0.001). Similarly, participants who had a monthly income or medical insurance, but it did not cover the treatment cost, showed significantly increased odds (OR: 1.22; 95% CI: 1.03–1.46, *p* = 0.024 and OR: 1.15; 95% CI: 1.03–1.29, *p* = 0.017, respectively) of self-medication. Furthermore, participants with chronic diseases showed significantly increased odds (OR: 1.40; 95% CI: 1.21–1.63, *p* < 0.001) of self-medication compared to those without chronic diseases (Table 6).

**Table 6:** Univariate and multivariate logistic regression analysis showing predictors of self-medication among study participants^b^

Dependent: SM prevalence		No	Yes	Univariate OR (95% CI) ^a^	Multivariate aOR (95% CI)
Age	Mean (SD)	24.7 (8.4)	26.2 (9.9)	1.02 (1.01–1.02, *p* < 0.001)	1.01 (1.00–1.02, *p* = 0.008)
Gender	Male	976 (37.1)	1657 (62.9)	–	–
	female	1874 (37.4)	3136 (62.6)	0.99 (0.89–1.09, *p* = 0.772)	1.06 (0.96–1.18, *p* = 0.257)
Area	rural	683 (38.6)	1087 (61.4)	–	–
	urban	2167 (37.0)	3695 (63.0)	1.07 (0.96–1.19, *p* = 0.217)	1.05 (0.93–1.18, *p* = 0.459)
Education level	unlearned	11 (18.3)	49 (81.7)	–	–
	preuniversity education	263 (36.9)	450 (63.1)	0.38 (0.19–0.72, *p* = 0.005)	0.50 (0.23–0.99, *p* = 0.057)
	university education	2318 (37.7)	3831 (62.3)	0.37 (0.18–0.69, *p* = 0.003)	0.60 (0.29–1.17, *p* = 0.156)
	high level	258 (35.8)	462 (64.2)	0.40 (0.20–0.76, *p* = 0.008)	0.61 (0.28–1.20, *p* = 0.170)
Governmental employee	no	2623 (38.2)	4252 (61.8)	–	–
	yes	227 (29.6)	541 (70.4)	1.47 (1.25–1.73, *p* < 0.001)	0.89 (0.70–1.15, *p* = 0.380)
Private employee	no	2595 (37.4)	4338 (62.6)	–	–
	yes	255 (35.9)	455 (64.1)	1.07 (0.91–1.25, *p* = 0.427)	0.79 (0.62–1.01, *p* = 0.060)
freelancer	no	2702 (38.4)	4332 (61.6)	–	–
	yes	148 (24.3)	461 (75.7)	1.94 (1.61–2.36, *p* < 0.001)	1.57 (1.22–2.03, *p* = 0.001)
Not working or retired	no	2389 (36.3)	4201 (63.7)	–	–
	yes	461 (43.8)	592 (56.2)	0.73 (0.64–0.83, *p* < 0.001)	0.61 (0.49–0.77, *p* < 0.001)
Medical field	no	1599 (36.1)	2827 (63.9)	–	–
	yes	1251 (38.9)	1966 (61.1)	0.89 (0.81–0.98, *p* = 0.014)	0.73 (0.59–0.90, *p* = 0.003)
Nonmedical student	no	2208 (36.4)	3862 (63.6)	–	–
	yes	642 (40.8)	931 (59.2)	0.83 (0.74–0.93, *p* = 0.001)	0.72 (0.58–0.90, *p* = 0.003)
Monthly Income	Prefer not to say	320 (36.5)	556 (63.5)	–	–
	no	164 (36.9)	280 (63.1)	0.98 (0.78–1.25, *p* = 0.885)	1.03 (0.80–1.32, *p* = 0.811)
	Yes, but it does not cover the treatment cost	480 (32.0)	1021 (68.0)	1.22 (1.03–1.46, *p* = 0.024)	1.38 (1.15–1.66, *p* = 0.001)
	Yes, and it covers the treatment cost	1885 (39.1)	2935 (60.9)	0.90 (0.77–1.04, *p* = 0.150)	1.02 (0.87–1.19, *p* = 0.812)
Medical insurance	No, I don’t have medical insurance	1647 (37.1)	2796 (62.9)	–	–
	Yes, but it does not cover the treatment cost	601 (33.8)	1175 (66.2)	1.15 (1.03–1.29, *p* = 0.017)	1.34 (1.17–1.52, *p* < 0.001)
	Yes, and it covers the treatment cost	602 (42.3)	821 (57.7)	0.80 (0.71–0.91, *p* < 0.001)	1.03 (0.89–1.19, *p* = 0.695)
Chronic disease	no	2562 (38.2)	4140 (61.8)	–	–
	yes	288 (30.6)	653 (69.4)	1.40 (1.21–1.63, *p* < 0.001)	1.22 (1.04–1.43, *p* = 0.015)

^a^*OR* Odds ratio, *CI* Confidence interval, *aOR* Adjusted odds ratio

^b^Test assumption (Hosmer-LEMESHOW: *p* value = 0.658 *p* value indicating the goodness of model fit)

In multivariate logistic regression, increasing age was also associated with a significant increase in self-medication odds (aOR: 1.01; 95% CI: 1.00–1.02, *p* = 0.008) having a monthly income or medical insurance that does not cover the treatment cost retained significantly increased odds with higher folds (aOR: 11.38; 95% CI: 1.15–1.66, *p* = 0.001 and aOR: 1.34; 95% CI: 1.17–1.52, *p* < 0.001, respectively). Additionally, participants with chronic diseases showed significantly increased odds (aOR: 1.22; 95% CI: 1.04–1.43, *p* = 0.015) of self-medication compared to those without chronic diseases (Table 6).

## Discussion

The uncontrolled practice of self-medication results in serious health hazards [41]. Its prevalence is high in Arab countries [42]. Many factors influence SM, including socioeconomic status, access to health care facilities, and the emergency of the condition, as previously reported [43]. The COVID-19 pandemic emphasized these factors and added fear of the infection, lockdown policies, and increased internet searches about self-medication [21]. As follows, the public became more liable to misinformation and misuse of medicines [44, 45]. This paper aimed to assess the prevalence, influencing factors of self-medication, the commonly used drugs, and the sources of information about SM among Arab countries during the COVID-19 pandemic. This study reported that self-medication practice was highly prevalent among respondents. The most commonly used drugs were analgesics, antipyretics, and vitamins. The common associated risk factors were experience with similar health conditions and the urgency of the problem. Pharmacist consultation was the most frequent source of information for self-medication. Predictors of self-medication practice were older age, having chronic diseases, and having monthly income or medical insurance that does not cover the treatment costs. It is considered the first one of its kind that included such a large sample size across the Arab region during the COVID-19 pandemic.

We found that 62.7% of the participants self-medicated during the COVID-19 pandemic; the prevalence rate ranged from 40.4% in Palestine to 72.1% in Egypt. These results differ from the findings of an earlier systematic review across the Middle East before the pandemic revealing prevalence rates ranging from 35.4% in Saudi Arabia to 83% in Iran [39]. Additionally, similar studies conducted in different countries around the world showed significant health hazards of SM [7, 46–54]. This study detected an increase in the consumption rate of SM during the pandemic in 19.6% of the self-medicated participants (SMPs), no change in 63.7%, and a decrease in 16.3%.

The data clarified a significant association of SM with chronic disease. Other studies revealed similar findings [55, 56]. The reason may be due to the effective use of medicines in previous similar conditions [57]. Experience with similar conditions was the most frequent reason for SM (74.6%) among SMPs. The stable character of chronic disease may also contribute to SM practice rather than visiting a physician [55]. Approximately 41.6% of SMPs reported that their reason for SM was fear of infection from health care units. This could be explained by the possibility that a lack of information regarding the COVID-19 disease may have evoked people’s worries and fears of catching it [8, 58, 59]. More than one-third (38.2%) of participants who consumed medicines thought that SM practice would protect them from COVID-19. Other causes of SM in the present study were urgency of the condition (47.2%), prior commitments and lack of time (33%), cost of the consultation (23.9%), and transport unavailability (10%). These reasons were consistent with those of previous studies [50, 54, 58].

The usage of certain drugs is consistent with COVID-19 symptoms; fever relief (65.1%), vitamins (57.1%), and antitussives (47.6%) were the most common drugs during the COVID-19 surge after painkillers (86%). These results were similar to those of previous studies [44, 46, 54]. Surprisingly, the reported SM practice not only included over-the-counter drugs but also prescribed drugs, specifically antibiotics (43.3%). This raises concerns as drug-resistant deaths are of considerable numbers, not to mention the economic burden in treating these cases [60].

In this study, the most frequent sources of information were pharmacist consultation (66.7%), academic knowledge (50.1%), and prior prescriptions for them (50.1). Previous studies agreed with our results [50, 61–64]. Hence, pharmacists play a key role in directing the population to appropriate SM [65]. However, some pharmacists seek profits, and their practice leads to inappropriate SM [66]. Strict policies and regulations should be applied to avoid these unethical behaviors.

The present data showed a significant increase in SM practice with increasing age, as reported earlier in Jordan, China, Nigeria, and Peru [7, 67–71]. This finding can be explained by the fact that elderly individuals tend to take care of their health to avoid aging-related diseases [69]. Some studies disagreed with these results and showed that SM practice was more common in younger individuals [72, 73]. Similar to Albawani, S. M. et al., we found that SM was not significantly associated with gender (male/female) or residence (urban/rural) [62]. However, these findings were in contrast with earlier studies [74–80].

Regarding the univariate logistic regression, we detected low odds of SM practice with preuniversity *(p = 0.005),* university *(p = 0.003),* and a high level of education *(p = 0.008)*. This may be due to the raised awareness among educated participants about the threats of inappropriate use of drugs; most of the participants were related to the medical field (42.1%) [77]. Amuzie, C. I. et al. showed a significant association between SM frequency and those who did not attain university education compared to those who did [69].

Government employees and freelancers were more prone to self-medication than other occupations. This may be due to the idea of visiting a doctor being time-consuming [74]. The current data showed that time commitments were the reason for SM for 33% of the self-medicated participants. Having no medical insurance was another reason reported by 69.79% of respondents. This might explain why they tended to self-medicate. In contrast, a study conducted in Ethiopia reported lower self-medication practices among governmental employees [72]. The authors observed low odds of SM among the nonworking and retired groups. The cause may be the low income to purchase medications [67]. Our results indicated that SM is significantly associated with having monthly income and medical insurance but did not cover the treatment cost. This was inconsistent with the findings of Shafie, Mensur et al., who showed high SM practices among the high-income group [56].

The findings of this study inform Arab healthcare policymakers about the status of self-medication in the Arab region. Hence, this may encourage the development of policies and regulations to control the inappropriate use of medications. Additionally, since pharmacists play an important role in informing people about SM, we recommend developing educational curricula for pharmacists that focus on the ethics of drug supply, holding frequent seminars to discuss these challenges, and posting professional ethics charts in pharmacies. Furthermore, health ministries should conduct frequent public awareness campaigns to educate the public about the negative impacts of medication misuse, to provide reliable sources of information about medications and to give advice on the appropriate use of nonprescribed medications.

The main strength of this study is the large sample size across ten countries with different sociodemographic data. The general population is another strength instead of previous studies that included specific populations: medical students, undergraduates, or elderly individuals. Hence, this wider scope helped in exploring the pattern of SM across a diverse variety of populations. Regarding the limitations, we did not add 10% to the sample size to cover the decrease in response. Additionally, selection bias was a possibility because only those with access to internet-connected smart devices could respond to the questionnaire. However, the authors allowed offline data collection in the countries with ethical approval. The convenience sampling strategy is also one of the limitations. Additionally, the estimated SM practice was from the start of the pandemic, which might lead to recall bias. We recommend further studies to assess awareness about SM and any associated health hazards in the Middle East.

## Conclusion

Due to the easy accessibility of drugs and the increased influence of social media during the COVID-19 pandemic, it became easier to practice SM inappropriately. The SM prevalence was slightly high during the COVID-19 pandemic. The most commonly used drugs were pain killers and fever-related medications. This made sense with the reported most frequent conditions, such as headache, common cold, and flu-like symptoms. Experience with similar conditions was the most common reason for SM, while pharmacist consultation was the most frequent source of information about SM. This phenomenon can be combated by increasing the public’s awareness of SM and its hazards, encouraging its wise use, promoting its rational consumption, and developing policies to restrict access to drugs other than over-the-counter drugs (OTCs).

## Supplementary Information

**Additional file 1.**

## Data Availability

All data generated during this study are included in this published article and the additional file.

## Abbreviations

OTC: Over the Counter
SM: Self-Medication
SMPs: Self-Medicated Participants
WHO: World Health Organization
