# Supplementary material for: Prevalence and influencing factors of self-medication during the COVID-19 pandemic in the Arab region: a multinational cross-sectional study
Source: BMC Public Health. 2023 Jan 27;23:180. doi: 10.1186/s12889-023-15025-y (PMC9880368; doi:10.1186/s12889-023-15025-y)
Supplement: Supplementary file 1 — Additional file 1. [file 12889_2023_15025_MOESM1_ESM.pdf]

Prevalence and influencing factors of self-medication practice during the COVID19 pandemic in the general population of the Arab countries.

The Self-medication practice is to use medicines to relieve symptoms or health conditions without consulting a physician

The objectives of this study are

-To assess the prevalence and influence of self-medication in the general population above 18 years old in the Arab countries

-To know the source of information and advice about SM.

-To determine the most common drugs used as SM.

-To detect the effect of demographic variables on SM promotion.

Privacy

No information that personally identifies the participants in this study will be collected

To participate in this research, you must be 18 years or older

Filling out this questionnaire constitutes your consent to use your answers for research purposes only without revealing your identity or personal data and you are free to withdraw at any time, without giving a reason, thank you.

.

Socio-demographic status

<table>
<tr><td>1. Age
(the answer must be a number)</td><td></td></tr>
<tr><td>2. Gender</td><td>o Male
o Female</td></tr>
<tr><td>3. The country in which you reside</td><td><table>
<tr><td>Egypt</td><td>Jordan</td><td>Saudi Arabia kingdom</td></tr>
<tr><td>United Arab Emirates</td><td>Bahrain</td><td>Sultanate of Oman</td></tr>
<tr><td>Yemen</td><td>Syria</td><td>Palestine</td></tr>
<tr><td>Algeria</td><td>Libya</td><td>Iraq</td></tr>
<tr><td colspan="3">Sudan</td></tr>
</table></td></tr>
<tr><td>4. The area you live</td><td>o Rural
o Urban</td></tr>
<tr><td>5. The Location of the area in which you live in the country</td><td>o In the North of the country
o In the South of the country
o In the East of the country
o In the west of the country</td></tr>
<tr><td>6. The educational level</td><td>o Uneducated
o Pre-university education (Literacy, technical or industrial education, preparatory, secondary….)
o University education /under-graduate
o Post-graduate degrees (M.A./Ph.D.)</td></tr>
</table>

7. The occupation status<br>(You can choose more than one answer)	☐ Governmental employee<br>☐ Private employee<br>☐ Freelancer or manual work<br>☐ Unemployed/ Job seeker/ retired<br>☐ Working in the medical field (including medical students)<br>☐ Non-medical student (Engineering, Commerce,……)<br>☐ Other (please specify)
8. Does your family's monthly income allow you to visit your physician if you need to?	○ No<br>○ Yes, but it does not cover the treatment cost<br>○ Yes, and it covers the treatment cost<br>○ Prefer not to say
9. Do you have medical insurance?	○ No, I don't have medical insurance<br>○ Yes, but it does not cover the treatment cost<br>○ Yes, and it covers the treatment cost
10. Do you suffer from a chronic disease?<br><br>(Chronic diseases such as diabetes, cardiovascular diseases such as hypertension, Respiratory diseases, Liver disease, Gastrointestinal diseases, cancer….)	○ Yes<br>○ No
**Self-medication practice**	
11. Have you ever taken any medication /drug without consulting a specialist doctor during the novel Coronavirus pandemic (COVID19)?	○ Never<br>○ Once<br>○ Seldom (2-3 times a year)<br>○ Sometimes (once every few months)<br>○ Often (once every few weeks)<br>○ Always
**Questions 12 to 16 are only for those who self-medicate during the COVID-19 pandemic.**	

12. Medicines/drugs you have taken without consulting a specialist doctor during the pandemic of COVID 19 (coronavirus infection) are<br><br>(((Please choose (Yes) or (No) in each of the following options))	

	Yes	No
Pain killers		
Fever-relieving medications (Antipyretic)		
Drugs for cough		
Anti-allergy		
Antibiotics		
Birth control pills		
Sleeping pills		
Vitamins		
Pills for indigestion		
Herbal/homeopathic		
Sedatives (to reduce irritability or anxiety)		
Other		

13. Illness conditions you have taken medication without consulting a doctor during the Coronavirus pandemic (((Please choose (Yes) or (No) in each of the following options))	

	Yes	No
Headache		
Pain elsewhere		
Fever		
Flu/ Cough/ Cold		
Diarrhea/constipation/vomiting		
Insomnia (Inability to sleep)		
Allergy		
Other		

14. The factors that have led you to the use of drugs without doctor consultation are (((Please choose (Yes) or (No) in each of the following options))	

	Yes	No
Prophylaxis (Protection) from the new coronavirus infection (COVID-19).		
The urgency of the problem		

	The high cost of the medical services		
	Prior commitments/ lack of time		
	Previous experience of similar problem and previous course of treatment		
	Unavailable/difficult transportation		
	Fear of catching the infection from hospitals or clinics		
	Other		
15. Your consumption of drugs without doctor consultation during the pandemic of Coronavirus (COVID 19) is	○ Decreased during the pandemic<br>○ As usual<br>○ Increased during the pandemic		
16. Sources of information that you rely on when taking medicines without consulting your doctor are<br><br>(((Please choose (Yes) or (No) in each of the following options))		Yes	No
	Prior prescriptions of my own		
	My own academic knowledge		
	Previous prescriptions of others		
	Counseling with the pharmacist		
	Friends and relatives		
	Advertisements		
	Internet		
	Medical and pharmaceutical students		
	Other		

Thanks for your time and effort.
